# Balancing the Risk of Cardiotoxicity Outcomes in Treatment Selection for Multiple Myeloma: A Retrospective Multicenter Evaluation of Ixazomib, Lenalidomide, and Dexamethasone (IRd) Versus Carfilzomib, Lenalidomide, and Dexamethasone (KRd)

**DOI:** 10.1002/jha2.70038

**Published:** 2025-04-28

**Authors:** Benjamin J. Lee, Michael Sayer, Ali A. Naqvi, Karen T. Mai, Pranav M. Patel, Lisa X. Lee, Aya F. Ozaki

**Affiliations:** ^1^ Department of Pharmacy Chao Family Comprehensive Cancer Center University of California Irvine Health Orange California USA; ^2^ Department of Clinical Pharmacy Practice School of Pharmacy & Pharmaceutical Sciences University of California Irvine California USA; ^3^ Division of Cardiology Department of Medicine University of California Irvine Health Orange California USA; ^4^ Division of Hematology Oncology Department of Medicine University of California Irvine Health Orange California USA

**Keywords:** cardiovascular, carfilzomib, ixazomib, multiple myeloma, proteasome inhibitor

## Abstract

**Introduction:**

Carfilzomib use has been extensively associated with cardiovascular toxicity; the risk with ixazomib, a novel oral proteasome inhibitor, is underreported and no large comparative analysis is available.

**Methods:**

We conducted a retrospective cohort study utilizing the TriNetX platform to compare toxicity outcomes among multiple myeloma patients who received lenalidomide, dexamethasone, and ixazomib (IRd) or carfilzomib (KRd).

**Results:**

After propensity‐score‐matching 478 patients from each cohort, the onset of new heart failure (HR 0.25; *p *< 0.001) and arrhythmias (HR 0.57; *p* = 0.014) at 6 months were significantly lower with IRd while overall survival at 3 years was similar (*p* = 0.50).

**Conclusion:**

IRd is associated with a significantly lower risk of cardiac toxicities compared to KRd.

## 1 Introduction

Multiple myeloma (MM) is a plasma cell dyscrasia that accounts for 10% of all hematologic malignancies in the United States (US) [1]. Proteasome inhibitors (PI) represent a significant advancement in the treatment of MM and have become first‐line therapy in most MM regimens. While the addition of carfilzomib to lenalidomide and dexamethasone (KRd) led to significant improvement in outcomes compared to historic cohorts among patients with relapsed/refractory MM (RRMM) and more recently, for newly diagnosed MM (NDMM) [2, 3, 4], the utilization of carfilzomib has been associated with significant cardiotoxicity. Increasing awareness of PI‐associated cardiac dysfunction has emerged in recent years as a significant and challenging treatment‐related adverse event (TRAE), especially given the predominance of septuagenarians and octogenarians in this patient population. With a median age of 69 years at diagnosis, of whom 65% are 65 years old or older [1], many MM patients have cardiovascular risk factors or comorbidities at diagnosis. With substantive evidence indicating persistent PI usage to confer survival benefit, the efficacy of PI‐based regimens must also factor long‐term tolerability [5, 6].

Ixazomib is a novel oral PI that was FDA‐approved in 2015 in conjunction with lenalidomide and dexamethasone (IRd) for the treatment of RRMM based on the Phase III TOURMALINE‐MM1 trial [7]. Studies among frail and transplant‐ineligible patients have also demonstrated positive outcomes with an acceptable toxicity profile in NDMM [8, 9]. Cross‐examination of clinical trials involving PIs suggests that cardiotoxic TRAEs are significantly lower with ixazomib versus carfilzomib, although no direct comparator studies have been performed. As the first and only oral PI, treatment with IRd also uniquely allows for an all‐oral triplet regimen that can be administered at home. Recent patient‐reported outcomes (PRO) data among MM patients highlighted the advantages of an all‐oral regimen and it being a high priority in their care [10]. In the present study, we aimed to compare the safety outcomes of the two common triplet backbones, IRd and KRd, in NDMM and RRMM patients, on a large scale.

## 2 Methods

We conducted a retrospective, multicenter cohort study utilizing the TriNetX platform [11], a global, federated research network that provides access to real‐time, de‐identified real‐world electronic health records. Adult patients (≥18 years of age) with an International Classification of Diseases, 10th Revision (ICD‐10) diagnosis code for MM (C90.0) who received triplet therapy with IRd or KRd at all US sites between January 1, 2016 and December 31, 2023 were evaluated for inclusion. Patients with any prior ixazomib or carfilzomib exposure were excluded from this analysis. Patients were also excluded if they had any documented history of heart failure (HF) (I50), long QT syndrome (I45.81), paroxysmal tachycardia (I47), atrial fibrillation and flutter (I48), or other cardiac arrhythmias (I49) in the past year. Primary endpoints included new‐onset HF and arrhythmia, which was defined as a composite endpoint including long QT syndrome (I45.81), paroxysmal tachycardia (I47), atrial fibrillation and flutter (I48), and other cardiac arrhythmias (I49), within the first 6 months of treatment. Secondary endpoints included new onset diarrhea and polyneuropathy at 6 months as well as all‐cause mortality at 3 years. Continuous and categorical variables were reported as mean (standard deviation) and count (percentage), respectively, as made available through TriNetX. Kaplan–Meier analysis was used to estimate cumulative risk of events of interest and overall survival (OS); between‐group differences were examined via the log‐rank test. Propensity score matching (PSM) utilizing a 1:1 nearest‐neighbor matching was performed to balance baseline characteristics. Matching covariates included patients’ demographic data (age, sex, race/ethnicity), cardiovascular comorbidities (hypertension, ischemic heart disease, dyslipidemia, type 2 diabetes mellitus, chronic kidney disease), and directed therapies (angiotensin converting enzyme inhibitors, angiotensin II receptor antagonists, beta‐blockers, and class I/III antiarrhythmics), as well as prior MM therapies (pomalidomide, bortezomib, daratumumab, cyclophosphamide, and melphalan). To account for toxicities associated with hematopoietic stem cell transplantation (HSCT) such as high‐dose melphalan conditioning, a subgroup analysis was performed among patients who did not proceed to HSCT during the 6‐month study period. Lastly, cardiovascular toxicities were compared between patients who received ixazomib 4.0 mg versus 3.0/2.3 mg in the IRd cohort to determine if a dose‐dependent effect was present. All statistical analyses were conducted utilizing the TriNetX Analytics platform.

## 3 Results

A total of 1534 patients were identified from the TriNetX database, 659 and 875 patients in the IRd and KRd treated cohorts, respectively. Patients who received IRd had a higher mean index age (65 vs. 61 years old; *p *< 0.001) and history of prior ischemic heart disease (9.3% vs. 5.8%; *p *= 0.130). A greater percentage of patients in the KRd cohort had higher previous exposure to bortezomib (42.1% vs. 22.2%; *p *< 0.001), cyclophosphamide (21.6% vs. 8.3%; *p *< 0.001), and daratumumab (25.3% vs. 5.3%; *p *< 0.001). The cumulative risk of HF, arrhythmia, diarrhea, and polyneuropathy at 6 months were 5.1%, 8.1%, 22.3%, and 8.1%, respectively, in the entire cohort.

**FIGURE 1:**
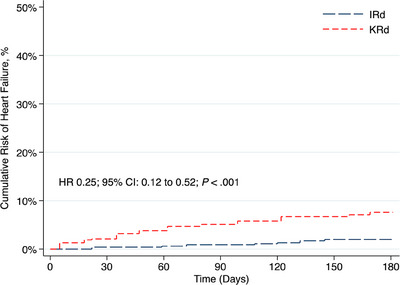
Cumulative risk of heart failure at 6 months after IRd versus KRd initiation. Abbreviations: IRd, ixazomib, lenalidomide, and dexamethasone; KRd, carfilzomib, lenalidomide, and dexamethasone.

**FIGURE 2:**
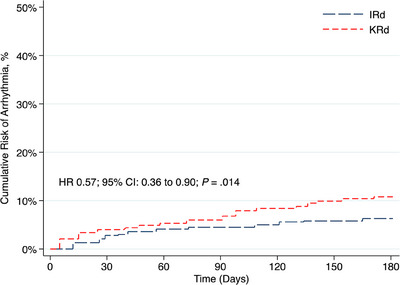
Cumulative risk of arrythmia at 6 months after IRd versus KRd initiation. Abbreviations: IRd, ixazomib, lenalidomide, and dexamethasone; KRd, carfilzomib, lenalidomide, and dexamethasone.

After PSM, 478 patients were included in each cohort. The mean index age was 63 years, 64.4% were identified as Caucasian, and roughly half of the patients were male (52.1%). IRd and KRd administration was similar among NDMM (23.8% vs. 25.8%) and RRMM (76.2% vs. 74.2%) patients (*p* = 0.50). Matched populations had no significant differences with respect to baseline demographics (Table 1). The cumulative risk of new‐onset HF at 6 months was 2.1% in the IRd cohort and 7.3% in the KRd cohort (HR 0.25; 95% confidence interval [CI]: 0.12–0.52, *p *< 0.001) (Figure 1). The cumulative risk of new arrhythmia was also significantly lower in IRd‐ versus KRd‐treated patients at 6 months (6.1% vs. 10.5%; HR 0.57; 95% CI: 0.36–0.90, *p* = 0.014) (Figure 2). Among RRMM patients, the risk of new‐onset HF (HR 0.58; 95% CI: 0.36–0.93; *p* = 0.021) and arrhythmia (HR 0.68; 95% CI: 0.48–0.96; *p* = 0.026) was also lower with IRd. There was no significant difference in the risk of new polyneuropathy (HR 0.69; 95% CI: 0.45–1.07; *p* = 0.106), while diarrhea was significantly lower in the IRd group (HR 0.73; 95% CI: 0.55–0.97, *p* = 0.027). A higher rate of HSCT did occur in KRd‐treated patients (16.5% vs. 6.8%), which may have contributed to the higher risk of diarrhea given the gastrointestinal toxicity associated with high‐dose melphalan. After excluding patients who received HSCT within the 6 months of triplet therapy initiation, risk of HF (HR 0.29; 95% CI: 0.13–0.68; *p* = 0.002) and arrhythmia (HR 0.55; 95% CI: 0.32–0.94; *p* = 0.028) remained lower with IRd while diarrhea was not significantly different (13.3% vs. 14.3%; HR 0.93; 95% CI: 0.59–1.45; *p* = 0.74). OS was similar in the matched cohorts including (76.2% vs. 78.3%; HR 1.10; 95% CI: 0.84–1.43; *p* = 0.50) and excluding (75.8% vs. 75%; HR 0.94; 95% CI: 0.70–1.26; *p* = 0.68) HSCT by 3 years. OS among RRMM patients was also similar between cohorts at 3 years (77.3% vs. 76.4%; HR 0.67; 95% CI: 0.72–1.24; *p* = 0.67). Among patients who received ixazomib 4.0 mg versus 3.0/2.3 mg in the IRd cohort, the cumulative risk of new‐onset HF (3.7% vs. 3.6%; *p* = 0.96) or arrhythmia (4.8% vs. 5.3%; *p* = 0.85) was no different at 6 months.

**TABLE 1:** Patient baseline characteristics after propensity score matching.

Variables^a^	IRd (*n* = 478)	KRd (*n* = 478)	*p*‐value
Male, No. (%)	252 (52.7)	249 (52.1)	0.85
Age (years)	63 ± 11	64 ± 10	0.82
Race/Ethnicity, No. (%)			
White	319 (66.7)	308 (64.4)	0.46
African American	88 (18.4)	87 (18.2)	0.93
Asian	13 (2.7)	14 (2.9)	0.85
Hispanic or Latino	33 (6.9)	32 (6.7)	0.90
Prior medical history, No. (%)			
Hypertension	195 (40.8)	192 (40.2)	0.84
Dyslipidemia	128 (26.8)	131 (27.4)	0.83
Diabetes mellitus	60 (12.6)	64 (13.4)	0.70
Chronic kidney disease	79 (16.5)	72 (15.1)	0.54
Ischemic heart disease	37 (7.7)	37 (7.7)	>0.99
Cerebrovascular disease	14 (2.9)	12 (2.5)	>0.99
Concurrent medications, No. (%)			
Beta‐blockers	98 (20.5)	97 (20.3)	0.94
RAS inhibitors	114 (23.8)	109 (22.8)	0.70
Treatment stage, No. (%)			0.88
Newly diagnosed MM	120 (25.1)	122 (25.5)	
Relapsed/Refractory MM	358 (74.9)	356 (74.5)	
Previously exposed chemotherapy, No (%)			
Pomalidomide	25 (5.2)	28 (5.9)	0.67
Bortezomib	125 (26.2)	132 (27.8)	0.61
Cyclophosphamide	54 (11.3)	50 (10.5)	0.68
Daratumumab	34 (7.1)	35 (7.3)	0.90
Melphalan	23 (4.8)	28 (5.9)	0.47

Abbreviations: IRd, ixazomib, lenalidomide, and dexamethasone; KRd, carfilzomib, lenalidomide, and dexamethasone; MM, multiple myeloma; RAS, rennin‐angiotensin system.

^a^ Reported as mean ± SD unless otherwise stated.

## 4 Discussion

In the present study, we show that MM patients receiving the triplet regimen of IRd versus KRd had a significantly lower risk of new HF and arrhythmia while there were no significant differences in incidence of polyneuropathy. Diarrhea was significantly lower with IRd; however, the higher rate of HSCT in the KRd cohort may have likely contributed, as a subgroup analysis of patients not proceeding to HSCT showed no difference. Additionally, our analysis showed no difference in cardiovascular toxicities between IRd patients who received the higher 4.0 mg versus lower 3.0 or 2.3 mg doses, indicating no increased risk with higher dosing.

As a wide array of treatment options are now available for patients with MM, treatment selection is heavily reliant on factors other than efficacy alone, including age, functional status, comorbidities, and patients’ expectations. As MM is an incurable neoplasm that mostly affects elderly patients, the focus of care is not only prolonging survival but also improving or maintaining quality of life. With increasing emphasis on PROs in supporting treatment decision‐making, management and prevention of TRAEs is being recognized as a high priority. Among TRAE associated with MM therapies, cardiotoxicity, diarrhea, and peripheral neuropathy have emerged as important toxicities. In a post hoc analysis of PROs in the E1A11 (ENDURANCE) trial evaluating bortezomib, lenalidomide, dexamethasone (VRd) compared to KRd, patients whose responses were categorized as high versus low “adverse event bother” were associated with early treatment discontinuation at 1 month (adjusted odds ratio [aOR] 2.20; 95% CI: 1.25–3.89), 2.8 months (aOR 3.41; 95% CI: 2.01–5.8), and 5.5 months (aOR 4.66; 95% CI: 2.01–5.80) [12]. Additionally, an all‐oral medication regimen has been identified as an important factor for MM patients due to greater perceived convenience and less disruption to daily life activities [10, 13]. In a retrospective longitudinal US claims data analysis, patients who received IRd had significant medical cost savings compared to those who received KRd, even after excluding treatment administration costs, suggesting an overall benefit for the US healthcare system [14]. Given the significantly lower risk of cardiotoxicity with IRd versus KRd in our analysis with comparable short‐term OS, greater consideration for utilizing IRd among MM patients should be made.

There are several limitations of our analysis. The extrapolated data from the TriNetX platform are not curated, thus bias could be present. Several key diagnostic parameters associated with cardiac function, such as echocardiogram or baseline B type‐natriuretic peptide (BNP), were not consistently available among all patients at set timepoints, so were not included in this analysis. Additionally, ixazomib dosing was not available for all included patients; however, as dose reductions are common with ixazomib utilization, initial dosing may not be reflective in characterizing outcomes and toxicities. Among patients with reported doses in the TriNetX database, cardiotoxicity outcomes were not different between those who received the higher 4 mg dose or the lower 3.0/2.3 mg dose. Carfilzomib dosing frequency (once vs. twice weekly) was also not reported within the TriNetX database. In order to strengthen our analysis, we balanced baseline risk between cohorts by excluding all patients with HF or arrhythmias within 1 year and via PSM. We also compared two common triplet regimens that differed only by the PI included. As numerous MM therapies, such as immunomodulatory agents or cyclophosphamide, can induce or compound cardiotoxicity, previous attempts at comparing cardiotoxicity of ixazomib versus carfilzomib alone may not accurately represent the true risk.

In conclusion, this large‐scale, multicenter database analysis shows that IRd has an improved cardiac profile compared to KRd for the management of MM. Future studies are needed to validate these findings over a prolonged treatment period as well as the impact of collaborative therapeutic monitoring for MM patients with cardiology services.

## Author Contributions

BJL, MS, and AFO conceptualized and designed the study, collected and analyzed the data, and wrote the initial draft of the manuscript. All authors interpreted the data and reviewed, critically revised, and approved the final version of the manuscript.

## Ethics Statement

The study was performed using the TriNetX database that uses de‐identified aggregate patient data for analysis. No protected health information or personal data are made available to the users of the platform. As a result, institutional review board approval was not required for this study.

## Conflicts of Interest

The authors declare no conflicts of interest.

## Clinical Trial Registration

The authors have confirmed clinical trial registration is not needed for this submission.

## Patient Consent Statement

All data reported in this study were obtained from the TriNetX database using de‐identified patient information compliant with HIPAA regulations. No personal information was obtained. Access to specific healthcare organization‐level data was not requested. No IRB approval was required. No patient consent was required.

## Data Availability

The data utilized for this study are available from TriNetX and restrictions apply to the availability of the data. Data can be made available at https://www.trinetx.com with the permission of TriNetX.

## References

[1] “Cancer Stat Facts: Myeloma,” National Cancer Institute, accessed April 21, 2022, https://seer.cancer.gov/statfacts/html/mulmy.html.

[2] A. K. Stewart , S. V. Rajkumar , M. A. Dimopoulos , et al., “Carfilzomib, Lenalidomide, and Dexamethasone for Relapsed Multiple Myeloma,” New England Journal of Medicine 372, no. 2 (2015): 142–152. 25482145 10.1056/NEJMoa1411321

[3] J. K. Jasielec , T. Kubicki , N. Raje , et al., “Carfilzomib, Lenalidomide, and Dexamethasone Plus Transplant in Newly Diagnosed Multiple Myeloma,” Blood 136, no. 22 (2020): 2513–2523. 32735641 10.1182/blood.2020007522 PMC7714092

[4] C. R. Tan , A. Derkach , D. Nemirovsky , et al., “Bortezomib, Lenalidomide and Dexamethasone (VRd) vs Carfilzomib, Lenalidomide and Dexamethasone (KRd) as Induction Therapy in Newly Diagnosed Multiple Myeloma,” Blood Cancer Journal 13, no. 1 (2023): 112. 37491332 10.1038/s41408-023-00882-y PMC10368661

[5] M. V. Mateos , P. G. Richardson , M. A. Dimopoulos , et al., “Effect of Cumulative Bortezomib Dose on Survival in Multiple Myeloma Patients Receiving Bortezomib‐Melphalan‐Prednisone in the Phase III VISTA Study,” American Journal of Hematology 90, no. 4 (2015): 314–319. 25557740 10.1002/ajh.23933

[6] A. Palumbo , F. Gay , F. Cavallo , et al., “Continuous Therapy versus Fixed Duration of Therapy in Patients with Newly Diagnosed Multiple Myeloma,” Journal of Clinical Oncology 33, no. 30 (2015): 3459–3466. 26282661 10.1200/JCO.2014.60.2466

[7] P. Moreau , T. Masszi , N. Grzasko , et al., “Oral Ixazomib, Lenalidomide, and Dexamethasone for Multiple Myeloma,” New England Journal of Medicine 374, no. 17 (2016): 1621–1634. 27119237 10.1056/NEJMoa1516282

[8] T. Facon , C. P. Venner , N. J. Bahlis , et al., “Oral Ixazomib, Lenalidomide, and Dexamethasone for Transplant‐Ineligible Patients With Newly Diagnosed Multiple Myeloma,” Blood 137, no. 26 (2021): 3616–3628. 33763699 10.1182/blood.2020008787 PMC8462404

[9] S. K. Kumar , J. G. Berdeja , R. Niesvizky , et al., “Safety and Tolerability of Ixazomib, an Oral Proteasome Inhibitor, in Combination With Lenalidomide and Dexamethasone in Patients With Previously Untreated Multiple Myeloma: An Open‐Label Phase 1/2 Study,” Lancet Oncology 15, no. 13 (2014): 1503–1512. 25456369 10.1016/S1470-2045(14)71125-8

[10] R. Ayto , O. Annibali , P. Biedermann , M. Roset , E. Sánchez , and R. Kotb , “The EASEMENT Study: A Multicentre, Observational, Cross‐Sectional Study to Evaluate Patient Preferences, Treatment Satisfaction, Quality of Life, and Healthcare Resource Use in Patients With Multiple Myeloma Receiving Injectable‐Containing or Fully Oral Therapies,” European Journal of Haematology 112, no. 6 (2024): 889–899. 38389468 10.1111/ejh.14180

[11] TriNetX , “Publication Guidelines,” TriNetX, 2019, https://trinetx.com/trinetx‐publicationguidelines/.

[12] J. D. Peipert , F. Zhao , J. W. Lee , et al., “Patient‐Reported Adverse Events and Early Treatment Discontinuation among Patients With Multiple Myeloma,” JAMA Network Open 7, no. 3 (2024): e243854. 38536173 10.1001/jamanetworkopen.2024.3854 PMC10973895

[13] E. Terpos , J. Mikhael , R. Hajek , et al., “Management of Patients With Multiple Myeloma Beyond the Clinical‐trial Setting: Understanding the Balance Between Efficacy, Safety and Tolerability, and Quality of Life,” Blood Cancer Journal 11, no. 2 (2021): 40. 33602913 10.1038/s41408-021-00432-4 PMC7891472

[14] L. Sanchez , A. Chari , M. Cheng , et al., “Comparison of Health Care Costs and Resource Utilization for Commonly Used Proteasome Inhibitor‐immunomodulatory Drug‐Based Triplet Regimens for the Management of Patients With Relapsed/Refractory Multiple Myeloma in the United States,” Journal of Managed Care & Specialty Pharmacy 29, no. 11 (2023): 1205–1218. 37776124 10.18553/jmcp.2023.23031 PMC10776283

